# Rapid adaptation to cold in the invasive cane toad *Rhinella marina*

**DOI:** 10.1093/conphys/coy075

**Published:** 2019-02-18

**Authors:** Cinnamon S Mittan, Kelly R Zamudio

**Affiliations:** Department of Ecology and Evolutionary Biology, Cornell University, Ithaca, USA

**Keywords:** cane toad, cold-tolerance, invasive species, phenotypic plasticity, rapid evolution, *Rhinella marina*

## Abstract

Understanding rapid adaptation to novel environments is essential as we face increasing climatic change. Invasive species are an ideal system for studying adaptation as they are typically introduced to novel environments where they must adapt if they are to persist. We used the invasive cane toad, *Rhinella marina*, to investigate the contribution of plasticity and evolution to rapid adaptation in a novel environment. *Rhinella marina* is a neotropical toad that has invaded areas with climates outside of its native environmental niche. The goal of this research was to understand how cane toads persist in northern Florida, the coldest region of their combined natural and invasive range, and originally thought to be beyond their thermal breadth. We measured Critical thermal minima in cane toads from the original, warm introduction location (Miami), and their northern range edge (Tampa) to determine whether northern toads were more cold-tolerant, and to examine the contribution of adaptive plasticity and evolution to any changes in tolerance. Our results show that following acclimation to cold temperatures, southern toads are less tolerant of cold than northern toads. This persistent population difference implies selection for cold-tolerance in northern populations. Differences in individual responses indicate that plasticity is also involved in this response. Our findings have implications for conservation because predatory cane toad invasions threaten local faunas, especially native amphibians. Characterizing specific adaptive mechanisms that allow *R. marina* to expand its range will identify evolutionary processes that shape a highly successful invasive species.

## Teaser

Invasive cane toads (*Rhinella marina*) in northern Florida experience the coldest temperatures anywhere in their native or invasive range. We test the thermal limits of toads from northern and southern Florida and find that northern toads are more tolerant of cold and have rapidly adapted to this novel environment.

## Introduction

How organisms respond to novel environments is a critical question in ecology and evolution. With climate change predicted to increase rapidly (Collins *et al.*, 2013), an understanding of whether organisms can persist, and through what mechanisms, has gained urgency. Adaptive plasticity, the ability of an organism to produce different phenotypes in different environments, is essential for organismal persistence during sudden climatic changes, but ultimately a species may need to evolve if it is to survive (Schlichting, 1986; Bradshaw and Holszapfel, 2006; Ghalambor *et al.*, 2007). Evolution and plasticity interact and determine species fates in new environments; however, despite considerable interest and decades of research, the relative contributions of plastic and evolutionary responses to rapid adaptation are still unresolved (West-Eberhard, 2003; Crozier *et al.*, 2008; Crispo *et al.*, 2010; Ghalambor *et al.*, 2015; Lande, 2015).

**Figure 1:**
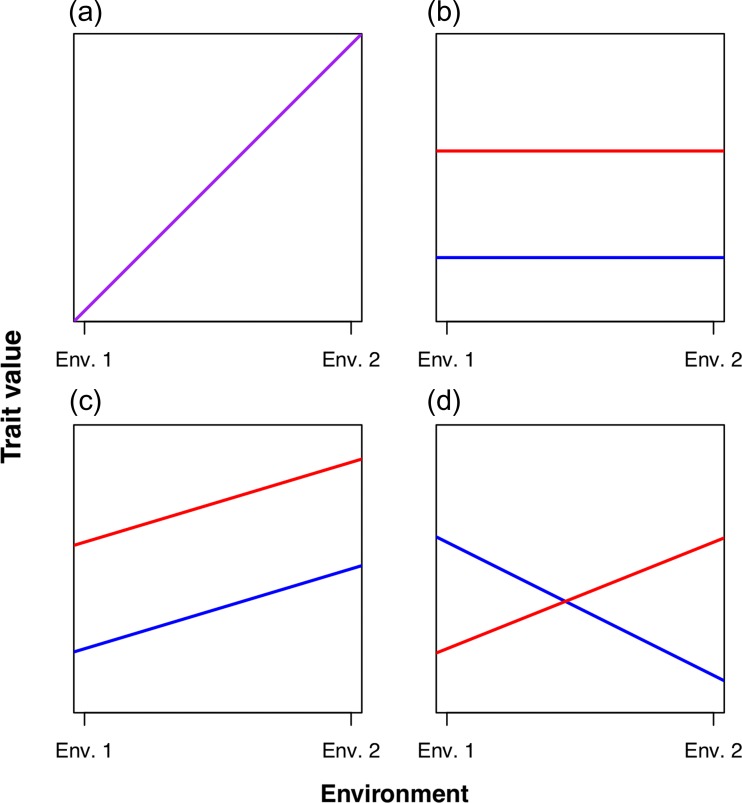
Example reaction norms with two genotypes tested in two environments. If each genotype is from a different population, differences in the reaction norms are evidence for adaptive divergence (evolution) or plasticity in the novel environment. **a.** Plasticity only. The trait values (intercepts) in each environment are equal, and plasticity (reaction norm slope) is the same. **b.** Evolution only. The genotypes have a difference in trait means, but do not exhibit plasticity for the trait. In **c.** Evolution of the trait mean with identical plasticity. The genotypes exhibit the same degree of plasticity, but a difference in trait means. **d.** Evolution in trait means and plasticity. The slopes of the lines, as well as their intercepts, are different.

One important question is the role of plasticity in rapid evolutionary response (DeWitt *et al.*, 1998; Ghalambor *et al.*, 2007). The first response to a novel environmental challenge is usually plastic; allowing the population to persist while selection shapes evolutionary response (Baldwin, 1896; Lande, 2015). However, several studies have uncovered the evolution of the plastic response itself over relatively rapid timescales (Kinnison and Hendry, 2001; Hairston and De Meester, 2008; Ghalambor *et al.*, 2015). While plasticity and rapid evolution in response to rapid change in environment are individually well documented, the occurrence of both trait mean evolution and the evolution of the plastic response are less frequently observed in the same system (Hairston and De Meester, 2008; Torres-Dowdal *et al.*, 2012; Stoks *et al.*, 2016). The two responses are not mutually exclusive; selection may result in simultaneous changes in both the trait mean and plasticity of the trait (Beaman *et al.*, 2016). For instance, a trait mean might increase overall, at the same time that the trait becomes more, or less (through genetic accommodation), sensitive to environmental cues (West-Eberhard, 2005). These two components are often visualized in a reaction norm, where a single genotype is measured across different environments in a common garden experiment (Handelsman *et al.*, 2012). The change in the response of an individual to the environmental stimulus—i.e. the slope of a reaction norm—is indicative of an evolutionary change in plasticity (Hairston and De Meester, 2008). In contrast, a change in the trait mean (height of the line) indicates evolution in the trait itself (Fig. 1) (Hairston and De Meester, 2008).

A second question is whether plasticity facilitates or slows rapid adaptation (West-Eberhard, 2003; Ghalambor *et al.*, 2015, 2007). Plasticity may facilitate adaptation by providing an immediate adaptive response, allowing the population to persist while selection shapes the evolutionary response (Baldwin, 1896; West-Eberhard, 2005). This initial adaptive plastic response may then become canalized through genetic accommodation, providing constitutive expression of a trait that was once a plastic response (Waddington, 1942; Pfennig *et al.*, 2010). Alternatively, adaptive plastic responses may slow the rate of evolution, as the underlying genetic variation for a trait will be masked by a plastic response that can achieve the trait optimum without selection (Ghalambor *et al.*, 2007). Several studies have approached this question by calculating the rate of evolution using Haldanes, a measure of trait evolution scaled by the standard deviation of the trait, in plastic systems, and comparing this to other examples of contemporary evolution (Räsänen *et al.*, 2003; Hairston and De Meester, 2008). Studies to date are inconclusive regarding the effect of plasticity on evolutionary rate, and few empirical studies exist (but see Hairston and De Meester, 2008; Schaum *et al.*, 2013), although one recent empirical study shows evidence that adaptive plasticity does in fact slow evolution (Ghalambor *et al.*, 2015).

One difficulty of addressing these questions in natural systems is that the initial plastic response can be transient; therefore it is best to observe plasticity in populations recently confronted with novel environmental pressures (Wellband and Heath, 2017). Invasive species, by definition, are exposed to novel environments at a discrete timepoint, and these environments can cause intense selection pressure over short periods (Yoshida *et al.*, 2007). Additionally, in invasive ranges with an abiotic gradient, such as elevation or temperature, the range can be strategically sampled to implement a space-for-time substitution design (Jueterbock *et al.*, 2016). Range-edge individuals have been exposed to a more extreme environment, while range-interior individuals have not. Thus, comparing the response of range edge individuals to the ‘ancestral’ response of the range-interior individuals, allows for inference about evolutionary response to the novel, range-edge environment.

**Figure 2:**
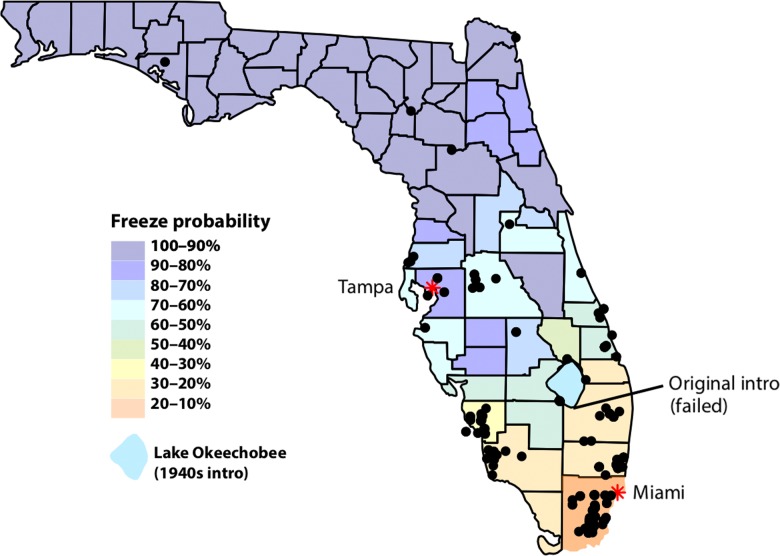
Cane toad sightings since 2010 (black dots, point data from EDDMapS, 2015). Counties are coloured by annual freeze probability (Southeast Climate Consortium, 2017), and red asterisks represent Tampa (Northern population) and Miami (Southern population).

**Figure 3:**
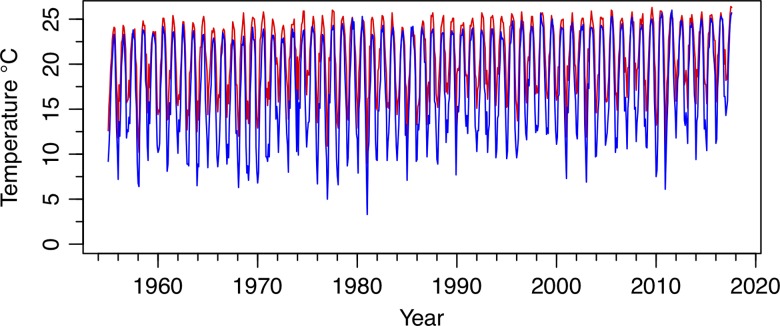
Temperature trends in Tampa (blue) and Miami (red) Florida from 1955 to 2017 (National Oceanic and Atmosphere Administration, 2017). Graph represents yearly temperature profiles for both localities based on the average low temperature for each month. Although localities are similar in summer, Tampa consistently has lower minimum temperatures during winter.

The cane toad, *Rhinella marina*, is native to Central and South America, but has established invasive populations around the world (Lever, 2001). The invaded regions vary in many environmental characteristics, and several instances of rapid trait evolution have been documented (Rollins *et al.*, 2015). *Rhinella marina* has invaded areas much colder than its native, tropical range, and previous research indicates temperature is a range-limiting factor in their invasions (Sutherst *et al.*, 2014). The coldest of these invasive ranges is in Florida, where intentionally introduced populations initially failed to establish due to winter mortality (Krakauer, 1968). The toads were later accidently released in a more southern and warmer location in 1955, where they established and spread across Florida, including the areas where introduction initially failed (Fig. 2; Easteal, 1981; EDDMapS, 2015). Tampa, Florida, at the northern range limit, consistently experiences cooler minimum temperatures than the introduction location in Miami, Florida (Fig. 3). In their native range, *R. marina* are active year-round, however, in Florida, their activity is restricted to March–October, presumably due to the thermal limits during the winter (Lever, 2001; Sutherst *et al.*, 2014). As cane toads must be mobile to eat and seek mates, we predict that *R. marina* populations in northern Florida have been selected for cold-tolerance, as well as endurance and locomotion during cold stress.

Here we used invasive *R. marina* populations in Florida to investigate the relative roles of plasticity and evolution in rapid adaptation. Our goals were two-fold: first we aimed to quantify differences in cold-tolerance between northern and southern populations in Florida, and second to characterize the relative contributions of plastic (Fig. 1a) and evolutionary response (Fig. 1b and c), and to characterize, if present, the evolution of plasticity itself (Fig. 1d). To test these questions, we measured critical thermal minimum (CTmin) in toads before and after acclimation to a control or cold temperature. CTmin is the temperature at which an organism loses motor response, and thus the temperature at which the individual would die if unable to escape (Cowles and Bogert, 1944). We also tested locomotor performance (speed and endurance) under cold stress. We predicted that we would find evidence for adaptive divergence, with northern toads exhibiting lower CTmin than southern toads, following acclimation. We also predicted that individuals would exhibit adaptive plasticity, manifested as an improvement in CTmin with cold acclimation, and that selection on plasticity would result in less variation in cold response in northern populations. Finally, we predicted that northern individuals would show improved locomotion in cold stress relative to southern toads after acclimation. Together, these analyses address how organisms at range edges, be they intentionally introduced or naturally expanding, adapt to novel selection pressures.

## Materials and methods

### Study system

Cane toads were intentionally introduced to Okeechobee, Florida to control cane beetles in the 1930s and 1940s, but failed to establish due to cold winter temperatures (Krakauer, 1968). Toads were then accidently released from a pet trade shipment in Miami in 1955, at which point they established locally (Krakauer, 1968). Sixty-two years and ~40 generations later, *R. marina* are now established in southern and central Florida, up to 90 miles north of Okeechobee. To assess possible adaptation at the northern range edge of this invasion, we collected toads at two localities in Florida, USA between 11 July and 20 July 2015. Southern toads were collected from Homestead, Florida (coordinates = 25.47N, –80.46E) and northern, range-front toads were collected in and around Tampa, Florida (coordinates = 28.03N, –81.94E and 28.06N, –82.39E). Twenty-eight individuals were collected from each locality and transported to Archbold Biological Station within 72 h of capture. Toads were transported in an air-conditioned car, and travel time was minimized (less than 3 h for both localities) to reduce their exposure to extreme temperatures.

To account for other factors that might affect individual variation in cold response, we recorded weight, sex, developmental stage and snout-vent-length (SVL) for each toad. For developmental stage, toads were classified as ‘juvenile’ or ‘adult’ based on SVL. Toads above 91 mm SVL were considered adults (McCoy *et al.*, 2008). We measured weight at initial capture, before the initial CTmin assessment, and before the final CTmin assessment. We marked toads individually by toe-clipping.

### Standard housing

All toads were housed in 59.2 × 47.5 × 31.2 cm plastic containers for the entirety of the experiment. Each tank had ~100mm of coconut substrate (Zoo Med EcoEarth), a plastic water container suitable for soaking, and cover provided for hiding. As *R. marina* are gregarious, toads were housed 3–4 per tank, and were separated by sex, and by size when possible. Toads were provided with food and water *ad libium* and held under 12-h light cycles. For lab acclimation and control samples, temperatures were held at 25°C, the preferred temperature of *R. marina* (Lever, 2001).

### Experimental acclimation

After 1 week of captivity acclimation at 25°C control temperature, tanks were split into control or experimental acclimation treatments. As toads in both localities were collected during the summer, temperatures were similar between the localities. 25°C was chosen because this is the preferred temperature of cane toads, and a temperature that both populations had likely been experiencing in the field over the past several months (Lever, 2001; National Oceanic and Atmosphere Administration, 2017). Toads were collected over a period of 10 days (see above), so this week-long acclimation to a control temperature was chosen to ensure that all toads had experienced an identical environment prior to experimentation. This strategy has been implemented in a similar study of this organism (McCann *et al.*, 2014). To keep temperatures as consistent as possible, tanks were transferred from the captivity acclimation room to a controlled temperature growth chamber. Tanks were held at either 10°C (experimental temperature) or 25°C (control temperature). The lower temperature (10°C) was chosen because it is the average minimum temperature in the coldest month in Tampa (National Oceanic and Atmosphere Administration, 2017). For the southern population, 15 toads were kept in the warm acclimation, and 12 in the cold acclimation. Due to a parasitic infection in one toad resulting in weight loss, one toad was removed from the study. For the northern population, 12 and 16 toads were included in the warm and cold acclimation treatments, respectively. Both cold (10°C) and warm (25°C) tanks were maintained simultaneously in the same growth chamber (Conviron® E7/2) in two different compartments. Due to the size of the growth chamber, only half the toads could be acclimated at any one time, thus we ran two sequential experimental trials. Toads from both populations were mixed between the two treatments, and between the two experimental trials. Tanks were rotated every other day, to avoid biases associated with tank position. Toads waiting for experimental acclimation were left in the captivity acclimation room at 25°C. Additionally, because some toads had a CTmin above the 10°C acclimation temperature, the acclimation temperature for the second trial was raised to 15°C. We included ‘trial’ as a variable in our linear regression testing the factors contributing to CTmin to account for differences in lab waiting time and cold acclimation temperature between the two experimental trials.

### CTmin

Toads were tested for CTmin after 1 week of captivity acclimation, and then again after 1 week of acclimation to experimental conditions. We chose to acclimate toads for 1 week at experimental temperatures because previous work showed that the period of time most relevant to CTmin response in *R. marina* was the temperature experienced 12 h before CTmin testing (McCann *et al.* 2014). Therefore, 1 week should be an adequate amount of time for toads to acclimate to the experimental treatment. CTmin was chosen because while it measures a biologically relevant response, it is sub-lethal and amphibians recover quickly from the assay (John-Alder *et al.*,1988). To measure CTmin, each toad was placed in an individual plastic container inside a cooler containing ice water following the protocol in McCann *et al.* (2014). To aid cooling, and to insulate the container from direct contact with ice as well as from changes in temperature due to brief openings of the cooler, each container was wrapped in a wet cotton cloth. Toads were chilled, undisturbed for 10 min, and then checked at 5-min intervals (McCann *et al.* 2014). At every 5-min checkpoint, each toad was tested for the self-righting response, the point at which a toad is too cold to right itself after being placed on its back, by flipping the toad upside down on a flat surface, and gently prodding the stomach. If the toad was still able to right itself, it was returned to the cooler and re-tested at 5-min intervals. Once righting response was lost, the body temperature of the toad was taken using a digital temperature probe inserted into the cloaca. Initial toad temperature, final toad temperature (CTmin), and time to CTmin were recorded and we calculated cooling rate for each toad in each trial. Because the cooler was periodically opened to remove and test toads, we did not record the air temperature of the cooler itself. On average, toad cooling rate was less than 1°C per minute, and in all cases was less than 1.5°C. This cooling rate was incorporated into statistical testing (Terblanche *et al.*, 2011). After temperature measurement, the toad was returned to its tank at 25°C for recovery.

### Locomotion

Because oxygen debt due to locomotor stress can persist for hours in amphibians we only tested locomotor ability once, after the final post-acclimation CTmin testing, so as not to influence CTmin results (Hutchison and Turney, 1975). As a consequence, we do not have plasticity data for locomotion traits. After the second measurement of CTmin (post-acclimation), toads were allowed to recover at 25°C for ~30 min, and then returned to their experimental temperatures. Toads were then re-acclimated to their experimental temperatures to ensure adequate time to re-pay oxygen debt, a process that could take up to 4 h (Gleeson, 1991) . Following 24 h of recovery, we tested for speed and endurance. All toads were tested in a 25°C environment (*N* = 55, population and acclimation numbers as in CTmin trials, above), and the skin temperature of the toad was taken before and after testing, to correct for any warming to ambient temperatures in the cold-acclimation toads. Toads were tested at 25°C because the 10°C acclimation chambers were not large enough for locomotion trials. We measured speed and endurance by starting the toads at one end of a walled-in racetrack (3m long × 30cm wide × 50cm high), and recording the time taken to reach the end of the track (McCann *et al.*, 2014). Speed was calculated by dividing three metres by the time taken to hop the three metres. To encourage toads to hop, each toad was gently prodded with a small paintbrush (McCann *et al.*, 2014). Endurance was measured as the number of hops taken by the toad before it stopped hopping for longer than 30 s. This 30-s exhaustion period was selected based on methods from McCann *et al.* (2014). Similar studies have used ten paintbrush taps with refusal to move as criterion for ‘exhaustion’; as we tapped the toads approximately once per second, our criteria gave toads longer to recover, and is thus a more conservative estimate of exhaustion (Llewelyn *et al.*, 2010). Following testing, toads were euthanized, and specimens deposited in the Cornell University Museum of Vertebrates (catalogue numbers a-0 016 126–a-0 016 241).

### Statistics

We tested for the effects of pre- or post-acclimation (‘Time Measured,’ a categorical variable indicating if the CTmin measurement was taken before or after experimental acclimation), population (‘Pop,’ North or South), and acclimation temperature (‘Acclim,’ warm or cold) on CTmin using a linear mixed effects model implemented in the lme4 package (Bates *et al.*, 2015) in R (v3.1.3, R Devolopment Core Team, 2015). ‘Trial,’ indicating whether toads were acclimated in the first or second experimental run, ‘Sex’ (Male, Female, Juvenile), weight (in grams), and cooling rate (degrees C/minute at which toads were cooled during each CTmin trial) were included as fixed effects, while ‘Tank’ (tank identity) and ‘ID’ (individual toad ID) were included as random effects. An interaction term between Time Measured, Pop and Acclim was also included. We checked the residual plots for all model assumptions.

Pairwise comparisons of treatment groups, Time Measured, and populations were also implemented in R using the lsmeans package, to determine differences between groups based on Time Measured, Acclim and Pop, while keeping other variables constant. Time Measured, Acclim, and Pop were included in this analysis, as they were significant in the fixed effects test. Statistics were corrected using the Holmes correction for multiple testing. Differences in the variance of the slope (reaction norm) between populations were tested using the variance test implemented in R.

To test differences in locomotion, we also used a Generalized Linear Mixed effects Model. We used the same explanatory variables as above for the modelling of CTmin. However, our response variables were speed (time to hop three metres), for the first model, and endurance (number of hops before exhaustion) for the second.

### Rate of evolution

The rate of evolution was determined by calculating Haldanes, which is the rate of evolution scaled to the standard deviation in the trait of interest. Here, we used the southern toad population trait mean for CTmin as the original time point, and the northern toad population CTmin mean as the current time point. Mean CTmin for each time point was calculated for cold-acclimated treatment toads only, using the following equation:

$$h=\left[\left(x_{2}{/s}_{p}\right)–\left(x_{1}{/s}_{p}\right)\right]/g$$

Where x_2_ is the mean trait value at current time point, x_1_ is the mean trait value at original time point, s_p_ is the pooled standard deviation of trait values, and g is generations (Hairston and De Meester, 2008). One generation is ~2 years in cane toads in temperate environments (Lever, 2001).

To test the significance of the Haldane value, we creating a null distribution as follows. We first randomly re-assigned cold-acclimated CTmin values to either the ‘North’ or ‘South’ group with replacement. We then re-calculated the Haldane value for each randomly assigned group. We repeated this 100 000 times, and then compared the actual Haldane value to this null distribution. Because our northern (*n* = 12) and southern (*n* = 16) measures of CTmin were unequal, we constrained the permutation to draw 16 and 12 individuals for the new groups, thus maintaining the empirical data structure.

## Results

### Populations differ in cold-tolerance

**Figure 4:**
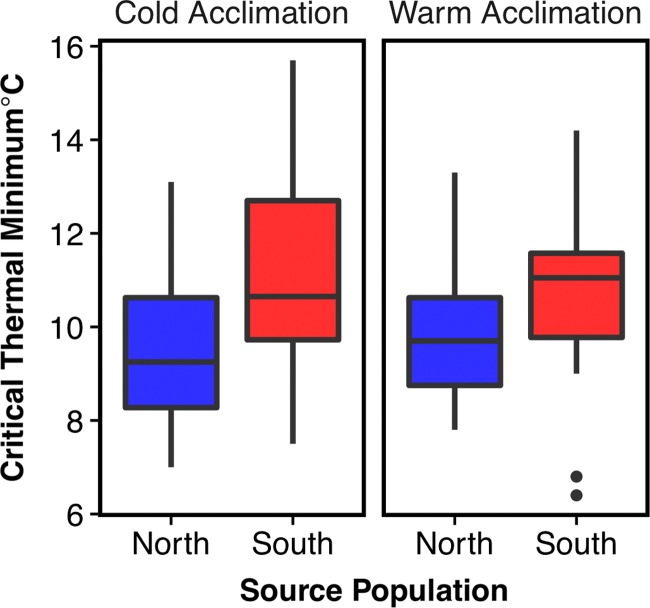
Population-level variation in CTmin. Each of the four boxplots represents a population and a treatment (i.e.—the first box on the left shows the northern, cold-acclimated treatment group). Whiskers extend to the minimum and maximum, with dots representing outliers. On average, northern populations that are cold-acclimated have lower CTmin than cold-acclimated southern populations (*t*(26) = –2.13, *P =* 0.04).

A Levene test showed that variance in CTmin was equal between Northern and Southern populations (*F*_(15,11)_ = 0.46, *P* = 0.15), so we proceeded with a *t*-test. In the fixed effects test of the variables incorporated in our GLMM, Time Measured (pre- or post-acclimation), Pop (population), and the two-way interaction between Acclim (acclimation temperature) and Time Measured were significant variables (*F*_(1,61)_ = 34.49, *P =* 1.9E–07, *F*_(1,48)_ = 8.84, *P* = 0.0046, and *F*_(1,61)_ = 8.31, *P* = 0.005, respectively) (Tables 1, 2). The fixed effects of Sex, Weight, cooling rate and Trial were insignificant (Tables 1, 2). A *t*-test showed that CTmin was significantly lower in northern versus southern populations (*t*(26) = –2.13, *P =* 0.04). Indeed, the median and mean CTmin within treatment groups were consistently lower for northern populations than southern populations (Fig. 4, Table 3).

**Table 1:** Generalized linear mixed model results

	Estimate	Std. Error	df	*t* value	Pr(>|*t*|)
(Intercept)	11.69	0.98	81.55	11.90	2.0E–16*
Time Measured A (after acclimation)	−2.65	0.61	61.99	−4.33	5.5E–05*
PopS (south)	1.79	0.68	72.46	2.62	0.01*
Acclim (warm)	−0.40	0.64	76.31	−0.62	0.54
Sex (juvenile)	0.21	0.78	47.52	0.27	0.79
Sex (male)	−0.41	0.46	47.24	−0.89	0.38
Weight	0.00	0.00	48.52	−0.08	0.94
Cooling rate	−1.01	0.67	73.58	−1.51	0.14
Round (2)	−0.17	0.38	51.08	−0.45	0.66
TimeMeasuredA:PopS	−0.30	0.64	50.28	−0.47	0.64
TimeMeasuredA:Acclim(warm)	1.76	0.76	56.54	2.31	0.02*
PopS:Acclim(warm)	−0.83	0.90	76.08	−0.92	0.36
CTmin.timeA:PopS:Acclim(warm)	0.11	0.91	50.33	0.12	0.91

The Time Measured (after acclimation), Population (south), as well as the iteraction of Time Measured and Acclimation are significant. Post-acclimation measurement lowers the CTmin estimate, while the interaction with the warm acclimation increases it. Population (south) also increases the estimate of CTmin.

**Table 2:** Fixed effects

	Sum Sq	Mean Sq	NumDF	DenDF	*F* value	Pr(>*F*)
Time Measured	48.53	48.53	1.00	61.27	34.49	<0.001*
Pop	12.44	12.44	1.00	47.59	8.84	<0.01*
Acclim	0.07	0.07	1.00	60.48	0.05	0.82
Sex	1.64	0.82	2.00	47.26	0.58	0.56
Weight	0.01	0.01	1.00	48.52	0.01	0.94
Cooling rate	3.20	3.20	1.00	73.58	2.27	0.14
Trial	0.28	0.28	1.00	51.08	0.20	0.66
Time Measured:Pop	0.42	0.42	1.00	50.33	0.29	0.59
Time Measured:Acclim	11.69	11.69	1.00	60.70	8.31	0.01*
Pop:Acclim	1.41	1.41	1.00	46.98	1.00	0.32
Time Measured:Pop:Acclim	0.02	0.02	1.00	50.33	0.01	0.91

Significance of fixed effects of the Generalized Linear Mixed Model (GLMM) with co-efficient estimate, degrees of freedom (numerator: NumDF, and denominator: DenDF) and *P*-value of each factor. The GLM model includes ‘Time Measured’ (before or after experimental acclimation), ‘Pop’ (population—North or South), ‘Acclim’ (acclimation temperature—warm or cold), ‘Sex’ (male, female or juvenile), ‘Weight’ (in grams), and ‘Trial’ (first or second experimental run) as fixed effects. We also included interactions between ‘Time Measured,’ ‘Pop,’ and ‘Acclim.’ ‘Time Measured,’ ‘Pop,’ and the interaction between Time Measured and Acclim are significant.

**Table 3:** Descriptive statistics for CTmin

	Mean (C)	Median (C)	Range (C)	N
Initial				
South	11.7	11.6	9.0, 15.7	27
North	10.5	10.3	8.0, 13.3	28
Warm Acclim				
South	10.1	10.6	6.4, 11.7	15
North	9.5	9.1	7.8, 12.0	12
Cold Acclim				
South	10.0	9.6	7.5, 13.0	12
North	8.7	8.3	7.0, 11.5	16

Mean, median, Range of CTmin and sample size for all groups. CTmin is lower in the northern toads in all groups.

In pairwise comparisons, the difference between CTmin before and after cold acclimation was significant in both northern and southern populations (Table 4). The difference in CTmin before and after warm acclimation (25°C control) was significant only in the southern population. The pairwise comparison between North and South in the cold post-acclimation treatment group was significant (*t*(73) = –2.18, *P =* 0.03) before correction for multiple testing, but marginally significant after (*t*(73) = –2.18, *P =* 0.06).

**Table 4:** Pairwise comparisons

Contrast	Estimate	SE	df	*t*-ratio	*P*-value	*P*-corrected
Initial,cold,N vs Acclimated,cold,N	2.65	0.61	61.99	4.33	<0.001	<0.001*
Acclimated,cold,N vs Acclimated,cold,S	−1.49	0.68	72.46	−2.18	0.03	0.06
Initial,warm,N vs Acclimated,warm,N	0.89	0.49	50.37	1.83	0.07	0.07
Initial,cold,S vs Acclimated,cold,S	2.96	0.66	60.29	4.49	<0.0001	<0.001*
Initial,warm,S vs Acclimated,warm,S	1.08	0.43	50.29	2.50	0.02	<0.05*

Pairwise comparisons of ‘Time Measured,’ ‘Acclim,’ and ‘Pop’ levels. Initial versus post-acclimated cold treatment groups were significantly different in both populations, as were initial versus post-acclimated warm treatment groups in the southern population. The difference between cold-acclimated northern individuals and cold-acclimated southern individuals was significant before Holme’s correction for multiple testing (*P =* 0.03), and marginally significant after (*P =* 0.06).

### All individuals exhibit plasticity

**Figure 5:**
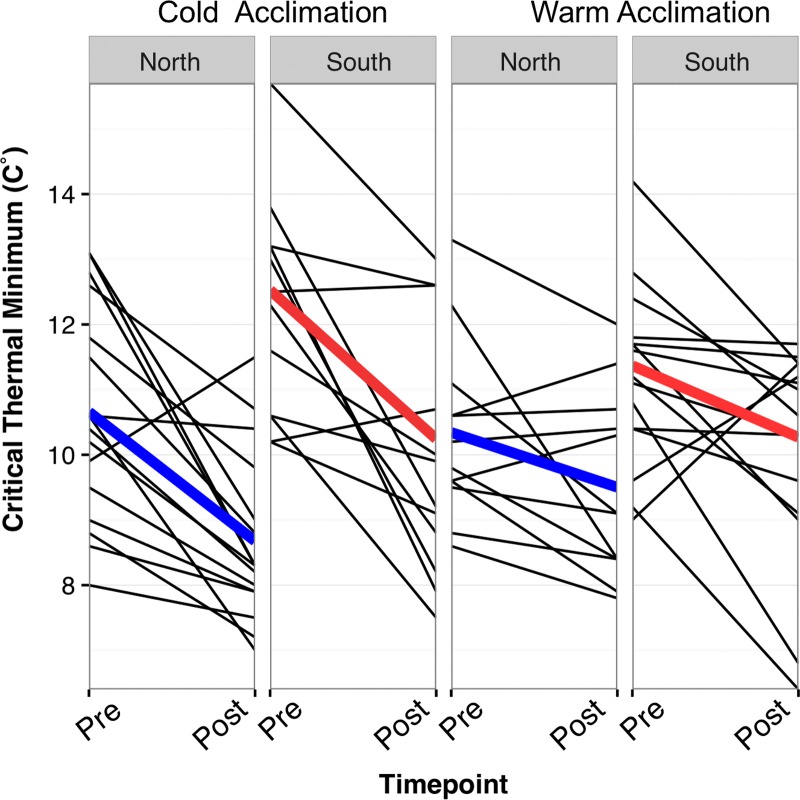
Individual reaction norms. Each line represents one toad measured for CTmin at two time points (*x*-axis): pre-acclimation and post-acclimation. Thick, coloured lines represent mean reaction norms within each treatment group.

All individuals showed some degree of plasticity (slope≠0), regardless of acclimation treatment (Fig. 5). All four mean reaction norms had a negative slope, indicating a lower post-acclimation CTmin (Fig. 5, bold lines). Within acclimation treatments, slopes were not significantly different between populations. Between acclimation treatments, slopes were significantly different, with steeper slopes in cold-acclimated treatment groups. This indicates that on average, cold-acclimated individuals were relatively more cold-tolerant compared to their initial CTmin assessment than warm-acclimated individuals.

At the population level, variation in plasticity (slopes in Fig. 5) was similar between populations, and the difference between populations insignificant (*F*_(15,11)_ = 0.54, *P* = 0.13, Fig. 5). In all groups, the reaction norm slope was negative (warm acclimated south = –1.1, warm acclimated north = –0.84 , cold acclimated south = –2.3 , cold acclimated north = –2.0) indicating average lower CTmins for the second CTmin test. Additionally, the range of cold-tolerance in cold-acclimated northern toads falls within the range of the cold-acclimated southern toads with the lowest CTmin thresholds (Fig. 5). Thus, although the average CTmin after cold-acclimation is lower in northern toads, a subset of toads in the south had comparable cold tolerances to those in the north.

### Locomotor performance is worse in cold temperatures

**Figure 6:**
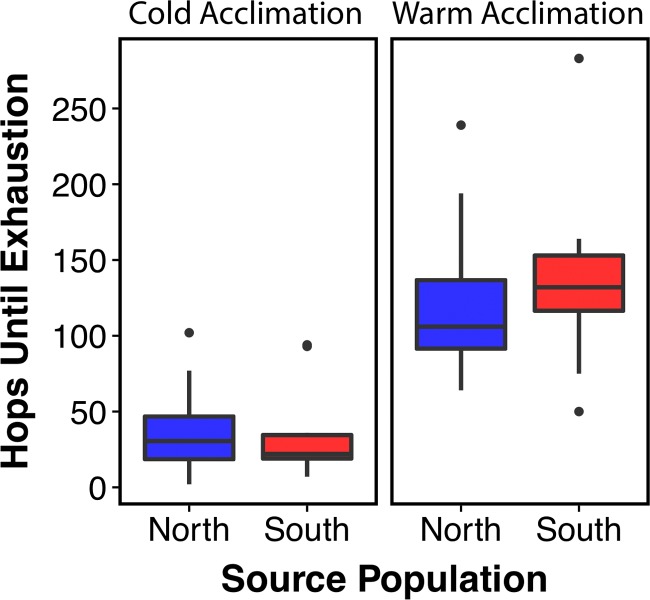
Population-level variation in locomotor performance following acclimation to warm or cold temperatures. Each of the four box plots represents a population and a treatment (i.e.—the first box on the left shows the northern, cold-acclimated treatment group). Whiskers extend to the minimum and maximum, with dots representing outliers. There is no significant difference between northern and southern toads within an acclimation treatment, but there is significant difference between treatment groups (*t*(54) = 5.11, *P =* 0.002).

We found no significant difference in locomotor performance between populations, however differences between treatment groups were significant (i.e. the variable ‘Acclimation’ was significant in all models. Total Hops: *t*(54) = 5.11, *P =* 0.002; Speed: *t*(8.7) = –2.633, *P* = 0.03) (Fig. 6, Supplementary Tables 1–5) Overall, toads kept in cold acclimation hopped significantly less, and significantly slower than toads kept in warm acclimation.

### Rate of evolution

We calculated the change in CTmin in northern versus southern toads to be –0.02 Haldanes. This negative value is in the predicted direction, indicating that CTmin decreased in northern toads, and the permutation test was significant (*P* = 0.04) (Supplementary Fig. 1).

## Discussion

The goals of this study were to assess adaptive divergence between southern and northern invasive cane toad populations, to decompose the response into plastic and evolutionary processes, and determine the role of plasticity in rapid adaptation. We predicted that due to the role of cool temperatures in determining *R. marina* range limits, as well as the initial failure of *R. marina* to establish in central Florida, that toads in northern Florida would show evidence of rapid adaptation to cold, mediated by both plastic and evolutionary responses. As predicted, northern cane toads are more cold-tolerant than their southern counterparts. Change in individuals over time and persistent population differences after acclimation indicate that this differential response is mediated both by adaptive plasticity and evolution. However, while there is ample variation in the reaction norms in both populations, it does not appear that the slope of the reaction norm (degree of plasticity) has changed overall. The cold-tolerance of northern toads, involving both adaptive plasticity and persistent differences from southern toads, suggests that *R. marina* will persist in this novel environment, and may continue spreading north in Florida.

Our data show that northern populations have a lower average CTmin than southern populations, even after cold acclimation, implying that northern *R. marina* are better adapted to the cold. One caveat is that the length of acclimation may affect results, though previous work has shown that the past 12 h of acclimation were a better predictor of CTmin performance in cane toads than the temperatures experienced in the previous month (McCann *et al.*, 2014). In addition, it is possible that some cold-tolerance may be due to maternal effects or other epigenetic effects (Mousseau and Fox, 1998). For instance, exposure to cold in the parents of the individuals tested may have resulted in epigenetic changes that mediate cold-tolerance in the offspring, rather than evolutionary changes *per se* (Sung and Amasino, 2004). Because the individuals used in this study were wild-caught, it is not possible to address this with our experimental design. To demonstrate rapid evolution more robustly, multiple generations of northern and southern *R. marina* must be tested.

Nonetheless, using a similar design, McCann *et al.* (2014), did not find persistent differences following acclimation in Australian *R. marina*, indicating that Florida *R. marina* are undergoing physiological adaptations distinct from what is ongoing in other invasions. The absence of persistent difference in CTmin in Australia may be due to the proximity between the two populations in the Australian study. This may allow individuals to travel between the two tested locations, preventing local adaptation to the cooler climate (McCann *et al.*, 2014). In contrast, northern and southern populations in Florida are separated by up to 320 km, and it is unlikely that the populations are interbreeding regularly. Thus, more limited gene flow in Florida may promote rapid local adaptation to cold at this invasion range edge.

This increase in cold-tolerance has implications for the potential range of *R. marina*. Currently, few studies take potential evolution into account when forecasting the spread of invasive species (but see Kolbe *et al.*, 2012), and it was originally thought that *R. marina* would not be able to spread to its current range (Easteal, 1981). These invasive populations appear to have underlying genetic variation that permits adaptation to cold, given the variation between individuals in this study (Fig. 5, panels 1 and 2), which could allow populations to become even more cold-tolerant through natural selection. However, the likely limit of cold tolerance will be freeze tolerance. While some amphibians do exhibit freeze-resistance, this requires a physiological mechanism of glucose production not present in *R. marina* (Storey and Storey, 1984). Thus, the evolution of an innovation such as freeze tolerance is less likely to occur. In addition, several studies have documented trade-offs in the evolution of thermal reaction norms, with selection on one extreme (i.e. CTmin), limiting tolerance at the other extreme (i.e. CTmax) (Angilletta *et al.*, 2003). Cane toads inhabit sub-tropical regions in Florida, thus maintenance of a high CTmax is likely an important limiting factor (Fig. 3). However, toads may also develop non-physiological cold-coping mechanisms, such as burrowing and the avoidance of freezing temperatures, which could also increase their temperate range (Easteal and Floyd, 1986). Future studies investigating habitat selectivity and buffering mechanisms would be especially useful for predicting further spread.

Our results underscore the role of plasticity during rapid adaptation to a novel environment. In this system, we can examine plasticity at individual and population levels. All individuals show some degree of plasticity with toads acclimated to cold showing the most improvement in CTmin, implying the presence of adaptive plasticity in response to extreme cold in most individuals. Studies of CTmax have also shown that longer-term acclimation improves physiological performance (Brattstrom and Lawrence, 1962).

Additionally, most individuals were more cold-tolerant the second time they were tested. In the warm acclimated populations, this implies that there is some physiological ‘memory’ of acute cold stress. This epigenetic phenomenon is well documented in plants and is a mechanism whereby a previous exposure to an environmental stress primes the organism to mount a more adapted response to the same stress in the future (Crisp *et al.*, 2016). This phenomenon, though less well documented, also occurs in vertebrates where it is called ‘hardening’ (Menke, 1982; Vo and Gridi-Papp, 2017). Studies of cold hardening in amphibians show that it is present even in tropical amphibians during novel cold stress (Vo and Gridi-Papp, 2017), and that when a test of CTmin is repeated, performance is better after a second exposure to cold stress (Menke, 1982). A difference in our study is that these effects persist for at least 1 week, while these previous studies re-tested after 1 h.

Cold-tolerance can be costly in vertebrates, with plastic response often requiring increased metabolic rate, increased enzyme production, and increased lipid metabolism (Gracey *et al.*, 2004). Indeed, a study in transgenic plants found that constitutive expression of cold-stress genes decreased plant fitness (Jackson *et al.*, 2004.). Therefore, an induced, rather than a constitutive response to cold in *R. marina* could reduce these investments, and explain the improvement triggered by the initial cold challenge.

At the population level, plasticity within treatment groups is the same (equal slopes) across populations (Fig. 5). This indicates no selection on the reaction norm itself, and that the trait means have changed in the north, without altering plasticity. This finding also implies that the response in both warm and cold environments have shifted by the same amount. This is in contrast to studies in which a change in the trait mean in the more challenging environment, without a change in the ancestral environment, results in a change in the reaction norm slope as a consequence (Hairston and De Meester, 2008). Thus, even when northern toads are acclimated to a warm temperature, they are still more cold-tolerant than warm acclimated southern toads. This difference from other studies could stem from the fact that northern toads are still experiencing both environments, with warmer summer temperatures more closely tracking the ancestral southern Florida environment (Fig. 3, National Oceanic and Atmosphere Administration, 2017). Therefore, selection in the novel environment (cold) is not decoupled from selection in the ancestral environment (warm).

We also predicted that the colder environment in the north would select for better locomotor performance during cold stress in northern toads. Our data show no significant differences between populations in locomotor performance; however, we found that cold-acclimated toads moved significantly less (Fig. 6). This demonstrates that the environmentally relevant test temperatures (10°C and 15°C), do significantly impact toad function. Therefore, cold temperatures in northern Florida likely severely limit toads’ ability to forage for food, a limitation not present in its tropical range. This limitation likely imposes strong selection pressure on toads to find ways to adapt (physiologically or behaviourally) to this novel stress.

Lastly, we calculated the rate of adaptive divergence of cold-tolerance for the northern toads in Florida. Historically, it was thought that adaptive plasticity could ‘shield’ genotypes from selection, thus precluding rapid evolution, but recent work confirms that the relationship plasticity and evolution is complex (for review see Ghalambor *et al.*, 2007). A growing number of studies have tested this empirically by calculating the rate of evolution in Haldanes in a plastic, evolving trait, and comparing this to other rates of known, rapidly evolving organisms (Kinnison and Hendry, 2001; Hairston *et al.*, 2005). Cold tolerance in northern cane toads has changed at a rate of –0.02 Haldanes (*P* = 0.04) (Supplementary Fig. 1), which is similar to rates uncovered in other rapidly evolving systems (Kinnison and Hendry, 2001; Räsänen *et al.*, 2003). Because we see significant divergence in this trait over a short time scale, plasticity does not appear to be impeding adaptation in cane toads (Crispo *et al.*, 2010; Ghalambor *et al.*, 2015; Lande, 2015).

Our results show that adaptation to cold in northern Florida cane toads is mediated by both plasticity, and by a persistent, adaptive divergence in tolerance between populations. Evidence for both plasticity and adaptive divergence in response to novel cold stress indicates that cane toads will be capable of further expanding their range. Variation in cold-tolerance within northern-populations also implies that there is further phenotypic variation on which selection can act. Nonetheless, toads are unlikely to establish much further north, where multiple days of sub-freezing temperatures will exceed their cold-tolerance. Thus far, cane toads in Florida are most abundant in heavily disturbed, urban environments and have not yet caused the ecological damage to native wildlife seen in other invasions(Smith and Larsen, 2012; Wilson and Johnson, 2018) . Like many invasions, cane toad populations in Florida have likely exceeded the threshold for total eradication but early detection efforts can be effective in preventing spread (Bogich *et al.*, 2008; Simpson *et al.*, 2009). Continued monitoring could prevent the spread of toads to northern Florida, and to more sensitive environments, avoiding the worst impacts seen in other invasions (Shine, 2014).

Future work in this system should quantify genetic variability of invasive and native populations, to further elucidate existing genetic variation, and adaptive potential. Work on the genetic architecture of Florida *R. marina* cold-tolerance would also clarify the genetic underpinnings of this rapid adaptation. Understanding the contributions of plasticity and rapid evolution to range expansions will be critical as we plan for global change and predict species’ fates under environmental stress.

## Supplementary Material

Supplementary Data
