# Supplementary material for: Rapid adaptation to cold in the invasive cane toad Rhinella marina
Source: Conserv Physiol. 2019 Feb 18;7(1):coy075. doi: 10.1093/conphys/coy075 (PMC6379050; doi:10.1093/conphys/coy075)
Supplement: Supplementary Data [file coy075_supplement.docx]

**Supplement**

**Supplementary Figure 1:**

Permutation test of Haldanes. Random permutation of the data show that the actual Haldane value calculated fell at *P =* 0.04 in the null distribution.

**
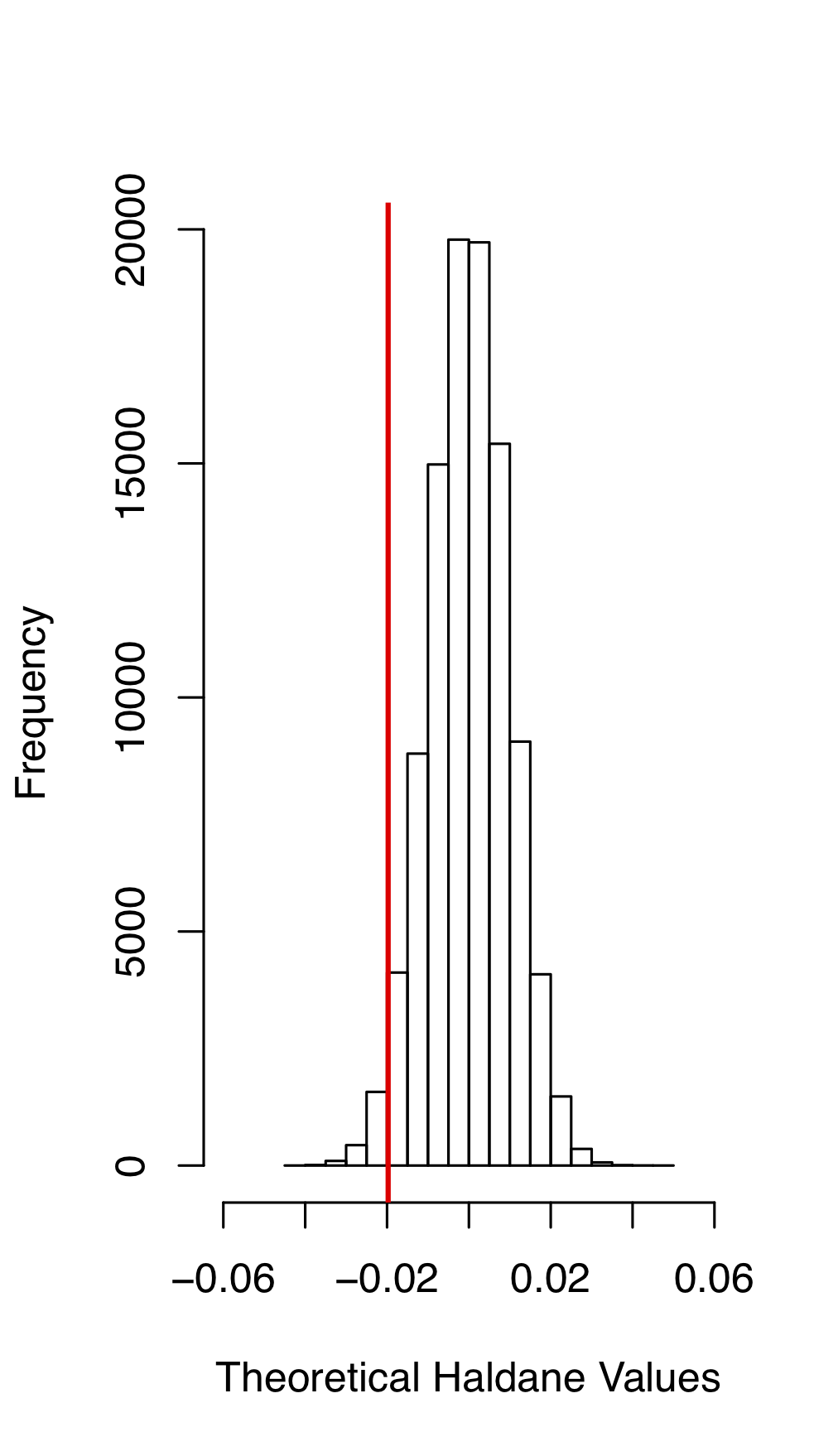
**

**Supplementary Table 1:** Descriptive data for locomotion (means across groups). Note: Two individuals from the south and one from the north refused to hop the full three-meter length. As speed was calculated as the time to hop three meters, these individuals do not have speed data. Additionally, three individuals from the north and three from the south are missing data for “Total Time” due to timer malfunctions during the trial. Sample sizes are given in parenthesis.

|  | Total Hops | Speed (m/s) | Total Time (sec) |
| --- | --- | --- | --- |
| Warm Acclim | | | |
| South | 136 (N=15) | 0.33 (N=15) | 152 (N=12) |
| North | 122 (N=12) | 0.35 (N=12) | 176 (N=11) |
| Cold Acclim |  |  |  |
| South | 34 (N=12) | 0.11 (N=10) | 132 (N=12) |
| North | 37 (N=16) | 0.12 (N=15) | 110 (N=14) |
| All |  |  |  |
| Warm | 130 (N=27) | 0.34 (N=27) | 163 (N=23) |
| Cold | 36 (N=28) | 0.12 (N=25) | 120 (N=26) |

**Supplementary Table 2:** Results of the Generalized Linear Mixed Model on total hops before exhaustion. Only the intercept and the effect of warm acclimation were significant.

|  | Estimate | | Std. Error | df | T value | Pr(>\|t\|) |
| --- | --- | --- | --- | --- | --- | --- |
| (Intercept) | 4.101354 | 0.787315 | | 9.57 | 5.209 | 0.000457* |
| PopulationS | -0.289681 | 0.299517 | | 8.04 | -0.967 | 0.36166 |
| Acclimwarm | 1.41666 | 0.276864 | | 6.41 | 5.117 | 0.001789* |
| Sexjuv | 0.086303 | 0.377642 | | 39.51 | 0.229 | 0.820412 |
| Sexmale | 0.374131 | 0.223951 | | 33.46 | 1.671 | 0.104132 |
| Weight | -0.002091 | 0.001808 | | 44.27 | -1.157 | 0.253679 |
| Round | -0.184822 | 0.188036 | | 5.83 | -0.983 | 0.364669 |
| PopulationS:Acclimwarm | 0.269443 | 0.392419 | | 6.3 | 0.687 | 0.516784 |

**Supplementary Table 3:** Significance of fixed effects incorporated into our GLMM for total hops before exhaustion. Acclimation temperature is the only significant variable.

|  | Sum Sq | Mean Sq | NumDF | DenDF | F value | Pr(>F) |
| --- | --- | --- | --- | --- | --- | --- |
| Population | 0.2249 | 0.2249 | 1 | 8.095 | 0.554 | 0.4777441 |
| Acclim | 26.7269 | 26.7269 | 1 | 6.315 | 65.8334 | 0.0001444* |
| Sex | 1.1912 | 0.5956 | 2 | 38.658 | 1.4671 | 0.2431426 |
| Weight | 0.543 | 0.543 | 1 | 44.271 | 1.3375 | 0.2536789 |
| Round | 0.3922 | 0.3922 | 1 | 5.83 | 0.9661 | 0.3646689 |
| Population:Acclim | 0.1914 | 0.1914 | 1 | 6.301 | 0.4714 | 0.5167837 |

**Supplementary Table 4:** Results of the Generalized Linear Mixed Model of speed (meters/second). Only the intercept and the effect of warm acclimation were significant.

|  | Estimate | Std. Error | df | T value | Pr(>\|t\|) |
| --- | --- | --- | --- | --- | --- |
| (Intercept) | 0.2062 | 0.1217 | 10.71 | 1.695 | 0.118955 |
| PopulationS | 0.0043 | 0.0477 | 10.79 | 0.09 | 0.930021 |
| Acclimwarm | 0.2528 | 0.0459 | 9.46 | 5.51 | 0.000315 * |
| Sexjuv | 0.0225 | 0.0411 | 42.27 | 0.549 | 0.586005 |
| Sexmale | -0.0194 | 0.0248 | 42.31 | -0.782 | 0.438459 |
| Weight | 0.0000 | 0.0002 | 38.25 | 0.042 | 0.966693 |
| Round | -0.0252 | 0.0318 | 8.66 | -0.792 | 0.449312 |
| PopulationS:Acclimwarm | -0.0348 | 0.0653 | 9.45 | -0.532 | 0.607021 |

**Supplementary Table 5:** Significance of fixed effects incorporated into our GLMM for speed (meters/second). Acclimation temperature is the only significant variable.

|  | Sum Sq | Mean Sq | NumDF | DenDF | F value | Pr(>F) |
| --- | --- | --- | --- | --- | --- | --- |
| Population | 0.000579 | 0.000579 | 1 | 10.222 | 0.1578 | 0.6994 |
| Acclim | 0.199802 | 0.199802 | 1 | 9.204 | 54.4561 | 3.721e-05* |
| Sex | 0.005427 | 0.002714 | 2 | 42.077 | 0.7396 | 0.4834 |
| Weight | 0.000006 | 0.000006 | 1 | 38.247 | 0.0018 | 0.9667 |
| Round | 0.002304 | 0.002304 | 1 | 8.657 | 0.6279 | 0.4493 |
| Population:Acclim | 0.001038 | 0.001038 | 1 | 9.453 | 0.283 | 0.607 |
